# Tic Frequency Decreases during Short-term Psychosocial Stress – An Experimental Study on Children with Tic Disorders

**DOI:** 10.3389/fpsyt.2016.00084

**Published:** 2016-05-17

**Authors:** Judith Buse, Stephanie Enghardt, Clemens Kirschbaum, Stefan Ehrlich, Veit Roessner

**Affiliations:** ^1^Department of Child and Adolescent Psychiatry, Faculty of Medicine, Technische Universität Dresden, Dresden, Germany; ^2^Department of Psychology, Technische Universität Dresden, Dresden, Germany

**Keywords:** tic disorders, Tourette syndrome, psychosocial stress, Trier Social Stress Test, free speech task, cortisol, skin conductance, heart rate

## Abstract

It has been suggested that psychosocial stress influences situational fluctuations of tic frequency. However, evidence from experimental studies is lacking. The current study investigated the effects of the Trier Social Stress Test (TSST-C) on tic frequency in 31 children and adolescents with tic disorders. A relaxation and a concentration situation served as control conditions. Patients were asked either to suppress their tics or to “tic freely.” Physiological measures of stress were measured throughout the experiment. The TSST-C elicited a clear stress response with elevated levels of saliva cortisol, increased heart rate, and a larger number of skin conductance responses. During relaxation and concentration, the instruction to suppress tics reduced the number of tics, whereas during stress, the number of tics was low, regardless of the given instruction. Our study suggests that the stress might result in a situational decrease of tic frequency.

## Introduction

Tic disorders (TDs) are neuropsychiatric disorders characterized by motor or vocal tics with regular first onset in childhood. Although the waxing and waning of tics over weeks and months is well known, its underlying pathophysiological mechanism is still obscure (1). The same has to be stated for mechanisms resulting in changes in a short-term perspective. Only few contextual factors, such as psychosocial stress, are suspected to be responsible for these fluctuations of symptoms (2–4).

There are a couple of studies investigating the relationship between stress (assessed *via* reports about life events or questionnaires on perceived stress) and fluctuations of tics in a longer perspective, i.e., over weeks or months. Early self-report-based studies suggested a relationship between life events and the onset or worsening of tics (5, 6). In line with this, a recent study found associations between several subscores of the Yale Global Tic Severity Scale (YGTSS) and major as well as minor life events (7). But the findings of Hoekstra et al. (8) are partly discordant, since only a minority of patients showed an association between tic severity and minor life events. While those studies focused on reports of life events, others examined the level of perceived psychosocial stress. The most compelling evidence for an association between perceived psychosocial stress and tics comes from a longitudinal study by Lin et al. (9) showing that overall levels of psychosocial stress were elevated in children and adolescents with TD compared to controls, and current levels of psychosocial stress were found to be a significant predictor of future severity of tics, obsessive–compulsive disorder (OCD), and depressive symptoms.

In addition to studies on tic fluctuations in a longer perspective, some studies focused on the effect of stress on tic frequency in a short-term perspective, i.e., in a specific situation. It has been shown that thermal stress leads to a marked situational increase of tic frequency (10, 11). Using a specific interviewing technique, O’Connor et al. (12–14) found that socializing was the situation in which tics appeared most likely. In another study on short-term fluctuation of tic frequency, the patients watched emotional scenes in a movie. Tic frequency was lower during emotionally charged scenes compared to baseline – particularly during happy and anger scenes. Interestingly, when asked later about emotional triggers for their tics, the patients reported that being happy was the only emotion which resulted in improvement of tics. A worsening of tics was attributed to anger by some of the patients, while others reported that anger did not affect their tics (15). A recent experimental study using a stress induction task indicates that psychosocial stress does not affect tic frequency *per se*, but psychosocial stress mainly reduced the ability to suppress tics, leading to an increase of tic frequency only in those situation with tic suppression (16).

Patients with TD also show an altered physiological stress response. They exhibited enhanced levels of cortisol secretion after exposure to psychosocial stress (17) and higher levels of adrenocorticotropin (ACTH) in blood plasma during lumbar puncture (18). Also, higher levels of corticotropin-releasing hormone (CRH) in the cerebrospinal fluid were found (19).

Up to now, there has been no study investigating the effect of psychosocial stress on short-term tic fluctuations in a larger sample size by using a standardized method to induce stress, by measuring physiological stress parameters to validate the stress induction, and by using objective measures of tic frequency at the same time. A detailed picture of the situational fluctuation of tic frequency, the (physiological) parameters modulating those fluctuations and the relationship between the patients’ subjective experience, and an objective measure of tics is a prerequisite of a successful behavioral therapy, e.g., with the well-established habit reversal training.

We aimed to elucidate these potential relationships by running an experimental design, in which we compared tic frequency during standardized induced stress vs. concentration vs. relaxation, and by combining measures of cortisol, heart rate, and skin conductance with self reports of psychosocial stress. Considering the suggestion that the relationship between tic frequency and stress is mediated by the ability to suppress tics, we also included a reinforced tic suppression condition in our study design.

## Materials and Methods

### Sample Characteristics

The participants were recruited in the TD outpatient clinic of the Department of Child and Adolescent Psychiatry of the TU Dresden. The sample consisted of 31 children and adolescents with either chronic tic disorder (*n* = 10) or Tourette syndrome (*n* = 21). The diagnoses were obtained according to the DSM-IV criteria in a clinical interview. Some of the patients also fulfilled diagnostic criteria of comorbid psychiatric disorders: OCD (*n* = 4), attention deficit hyperactivity disorder (ADHD) (*n* = 6), oppositional defiant disorder (*n* = 2), enuresis (*n* = 2), anxiety disorder (*n* = 1), adjustment disorder (*n* = 1), and insomnia (*n* = 1). Three patients were currently taking medication to treat their tics (aripiprazole *n* = 2 and tiapride *n* = 1), two patients were currently treated with ADHD medication (methylphenidate *n* = 1 and atomoxetine *n* = 1). The patients were aged between 7 and 17 years (mean 11.9 years), 26 of them were males and five females, respectively. The mean total tic severity score on the YGTSS was 14.13 (SD = 6.32), the mean motor tics score was 10.10 (SD = 3.59), and the mean vocal tic score was 4.03 (SD = 4.83).

### Task

Within the experiment, we simulated three situations (stress, concentration, and relaxation) with different levels of arousal. In addition, we gave two different instructions regarding the suppression of tics (reinforced volitional tic suppression and no suppression of tics).

#### Stress

Stress was induced by a free speech task similar to the first part of the children version of the Trier Social Stress Test [TSST-C; Buske-Kirschbaum et al. (20)]. The patients received the beginning of a story (in written form) and were told to finish the story as exciting as possible in front of a committee, which was announced as experts in judging the quality of children’s stories. After receiving the beginning of the story, the patients were given 5 min to think of an ending for the story and prepare for the speech in front of the committee. Thereafter, the patients were asked to stand in front of a table with the committee seated behind, consisting of two persons wearing white physician’s coats. The patients were then requested to finish the story in a free speech of 5-min duration. In order to increase the stress induction, the participants received no or only very little verbal and non-verbal feedback. Whenever patients finished the story in <5 min, they were stonily asked to continue. At the end of the whole experiment, the participants were debriefed about the actual aim of the task.

#### Concentration

In order to induce a concentrated state in the patients, we used a modified version of the symbol search task taken from the Hamburg-Wechsler-Intelligenztests für Kinder [HAWIK; Petermann and Petermann (21)]. The adaptation of the instruction served to provoke concentration only. Instead of provoking concentration and stress at the same time.

#### Relaxation

In the relaxing situation, the patients leaned back in a comfortable chair and listened to two pieces of quiet instrumental music composed by the italic composer Ludovico Einaudi (“Giorni Dispari” and “Fuori dal mondo”) *via* headphones.

#### Volitional Tic Suppression

In the tic suppression condition, the patients were instructed to suppress their tics as much as possible. To increase the motivation to do so, a 20-cent reward was promised for each tic-free interval of 30 s and disbursed in the end. For this purpose, the actual video was displayed online on a screen in an observation room, where a second investigator made a quick count of the number of tics.

#### No Suppression of Tics

In those conditions, the patients were instructed to “tic freely.”

### Procedure

The patients arrived in the early afternoon. The patient’s parents were completely informed about the procedure and the purpose of the study. The patients were informed about the procedure. They were not informed about the purpose of the stress induction task until debriefing at the end of the experiment. Written informed consent was obtained from both the participants and their parents. The study was approved by the ethics committee of the TU Dresden and was carried out in accordance to the approved protocol and the Declaration of Helsinki.

The patients were video recorded throughout the experiment from a camera in front of them. Vocal expressions were recorded.

The experiment followed a 3 situation × 2 instruction design, resulting in six experimental conditions: (1) relaxation + no suppression of tics, (2) relaxation + tic suppression, (3) concentration + no suppression of tics, (4) concentration + tic suppression, (5) stress + no suppression of tics, and (6) stress + tic suppression. Over the course of the experiment, each patient underwent all six conditions. The order of the conditions was randomized. We aimed for a full randomization. However, due to drop outs and technical difficulties, some sequences were overrepresented. We therefore checked for sequence effects statistically, as further described in Section “Statistical Analysis.”

**Figure 1:**
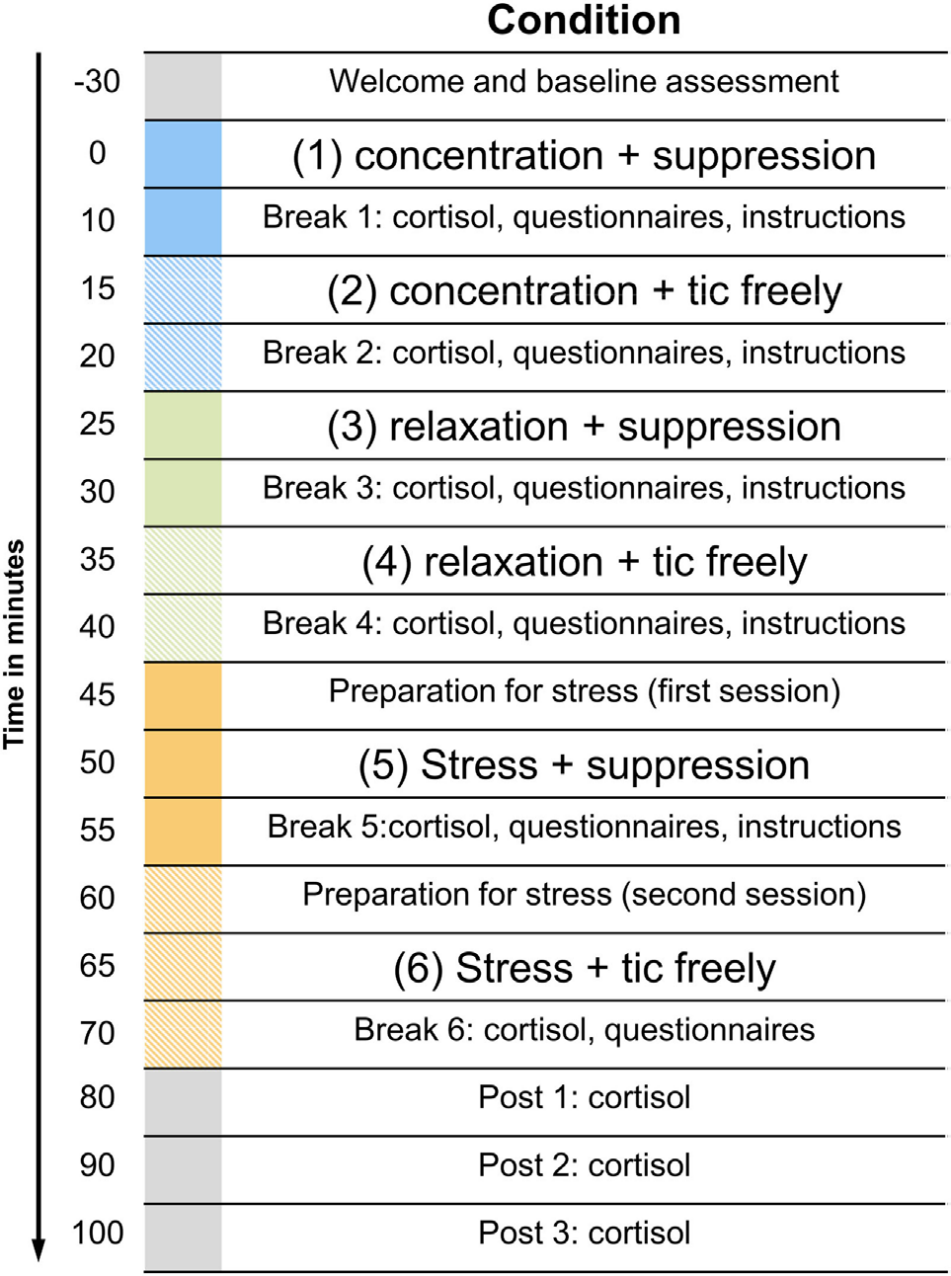
**Illustration of the experimental procedure**. The figure displays only one of the possible sequences. The order of the three situations with the different levels of arousal as well as the order of the instructions within these situations was randomized.

The duration of each condition was 5 min. Between the conditions, there was a 5-min break, in which cortisol samples were taken and instructions for the next condition were given. In addition, the participants answered three very short questionnaires during that break (see next section). To await the decrease of the cortisol response during the stress induction task, the break after the stress conditions was 30 min long. The experimental procedure in one of the possible variations is illustrated in Figure 1.

### Measures

Tics were coded offline from the video and audio recordings obtained throughout the whole experiment by two well-trained raters who were blind to the study hypothesis. Five data sets were coded by both raters independently in order to determine the inter-rater agreement. Since the inter-rater agreement was satisfying (80%), the remaining data sets were coded by only one of the raters. The coding was done with the software The Observer^®^ XT (Noldus).

Several physiological measures were obtained in order to determine the stress response to the different conditions: salivary cortisol, heart rate, and skin conductance.

Salivary cortisol was sampled using the Salivetten^®^ device (Sarstedt, Nümbrecht). This device is a small cotton swab, which has to be chewed for 30–60 s. The saliva samples were obtained at the end of each condition. In addition, the delayed increase of the cortisol concentration in response to the stress induction task was captured with three subsequent saliva samples taken 10, 20, and 30 min after the stress condition. The cortisol concentration was analyzed from the clear supernatant of the saliva with a chemiluminescence assay (CLIA, IBL-International, Hamburg). The cortisol concentration in the saliva was indicated in nanomoles per liter.

The heart rate was measured continuously with three electrodes positioned on the upper body and recorded with the BrainVision Recorder software (Brain Products GmbH). The data preprocessing (segmentation, baseline correction, and detection of *R* peaks) was done offline with the BrainVision Analyzer software (Brain Products GmbH). The final analysis was run with the Kubios Heart Rate Variability Analysis software. For each condition, an average score of the heart rate in beats per minute (bpm) was determined.

As another indicator of physiological arousal, we measured skin conductance with two resuable Ag/AgCl electrodes on the index and middle finger of the non-dominant hand. The skin conductance data were analyzed with the Matlab-based software Ledalab V3.4.7. After preprocessing (downsampling to 10 Hz and adaptive smoothing), continuous decomposition analysis was used to decomposed the data into continuous phasic and tonic components (22). For each experimental condition, the number of skin conductance responses (NSCR) was extracted. The threshold for detecting significant skin conductance responses was 0.01 μS.

The affective reaction to the previous condition was assessed with a couple of self-report questions. The Self-Assessment-Manikin Scale [SAM; Bradley and Lang (23)] was used to determine how much pleasure and arousal the participants experienced in the previous condition. A short version of the Perceived Stress Scale [PSS-4; Cohen et al. (24)] was applied to assess the subjective perception of psychosocial stress in the previous condition.

In addition, the Premonitory Urge for Tics Scale [PUTS; Woods et al. (25)] was used to assess the strength of premonitory urges in the previous condition. On that behalf, the original PUTS was modified into asking the participants, explicitly how they felt about their premonitory urges in the preceding situation.

### Statistical Analysis

Statistical analysis was done with IBM SPSS Statistics 21 Software. The two-way repeated measures analysis of variance (ANOVA) was conducted in order to analyze the effects of situation (stress vs. concentration vs. relaxation) and instruction (tic suppression vs. no suppression) on the number of tics, on the different physiological stress measures, and on the affective ratings. Before each ANOVA, Mauchly’s tests were computed to test the assumption of sphericity. Whenever the assumption had been violated, degrees of freedom were corrected using Greenhouse–Geisser estimates of sphericity.

In order to check for sequence effects, we ran additional repeated measures ANOVAs including the between-subject factor “sequence of the conditions.” We found no constant influence (main effects of sequence, interaction effects between sequence and situation, or interaction effects between sequence and instruction) on the number of tics or on any of the physiological stress measures (salivary cortisol, heart rate, and NSCR).

The findings reported in Section “Results” refer to the ANOVAs without “sequence of the conditions” as between-subject factor.

## Results

The mean raw scores for salivary cortisol, heart rate, and NSCR are listed in Table 1. The results of the ANOVAs for the different dependent measures are described in the following.

**Table 1:** **Number of tics, salivary cortisol level, heart rate, and skin conductance in the *n* = 6 conditions**.

		Number of tics (*N* = 28)	Cortisol (*N* = 20)	Heart rate (*N* = 26)	Skin conductance (*N* = 25)
Relaxation	No suppression	17.11 (18.28)	7.55 (3.22)	83.51 (8.88)	92.64 (36.67)
	Tic suppression	9.54 (12.53)	7.73 (3.98)	82.27 (9.14)	90.84 (34.55)
Concentration	No suppression	13.43 (14.84)	6.01 (2.28)	86.85 (10.43)	89.76 (23.55)
	Tic suppression	7.86 (9.80)	6.16 (2.25)	86.02 (10.06)	91.68 (22.42)
Stress	No suppression	8.29 (16.40)	11.06 (10.16)	100.21 (12.15)	113.60 (20.3)
	Tic suppression	7.89 (17.00)	11.46 (9.31)	98.89 (12.38)	120.56 (20.6)

*Tics: number of tics (duration of each condition: 5 min); cortisol: cortisol concentration in saliva in nanomoles per liter; heart rate in beats per minute; skin conductance: number of skin conductance responses (threshold = 0.01 μS). *N* indicates the number of participants, of whom the respective measurement were valid. Values are means (SD)*.

### Number of Tics

**Figure 2:**
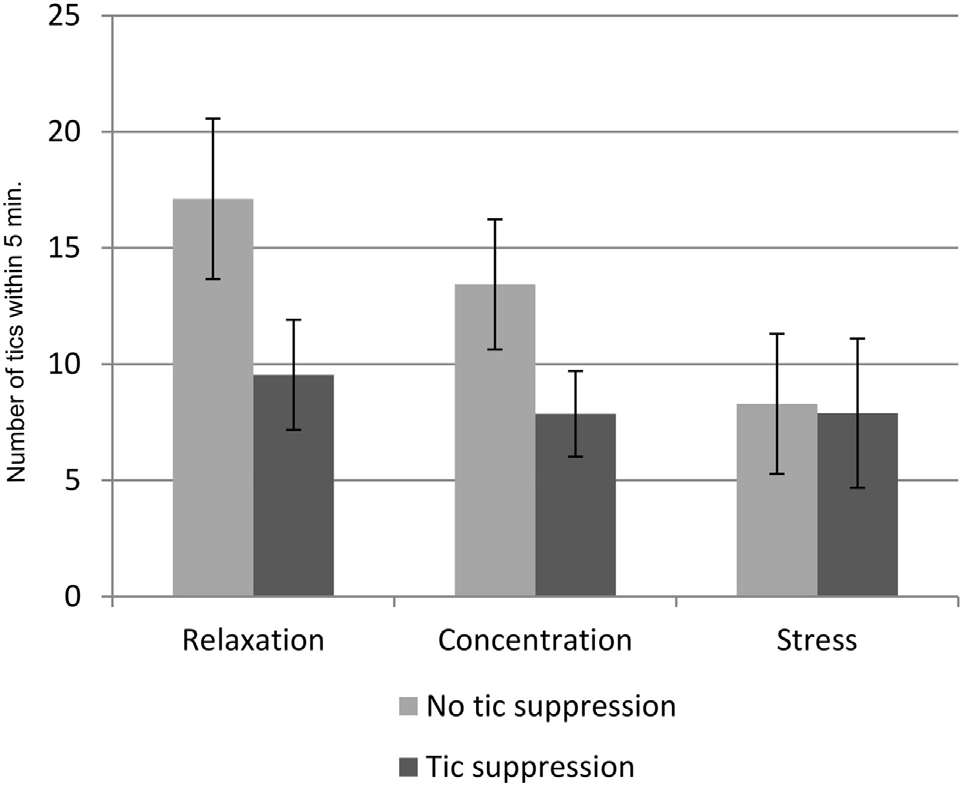
**Interaction effects of situation, and instruction on the number of tics**. Mean number of tics in the *n* = 6 conditions of 5 min duration each. Error bars indicate the SEM.

The average numbers of tics in the different experimental conditions are listed in Table 1. The main effect of situation on the number of tics was not significant, but reached trend level [*F*(2,54) = 3.1, *p* = 0.053]. *Post hoc* tests revealed a lower number of tics during stress compared to relaxation (*p* = 0.017), while there was no difference between stress and concentration or between concentration and relaxation. Instruction had an effect on the number of tics, indicating that the number of tics was reduced when the participants were instructed to suppress their tics [*F*(1,27) = 17.0, *p* < 0.001]. There was also an interaction effect between the factors situation and instruction [*F*(2,54) = 3.1, *p* < 0.044]. This interaction effect is illustrated in Figure 2.

### Cortisol Concentration in the Saliva

There was a main effect of situation [*F*(2,25.25) = 8.55, *p* = 0.004] on the salivary cortisol level. Instruction had no effect on the salivary cortisol level, and there was no interaction between the factors situation and instruction. However, in the case of the cortisol data, the assumption of normal distribution was not fulfilled, which makes the use of the two-way repeated measures ANOVA invalid. We therefore conducted separate non-parametric tests to analyze the effect of situation and the effect of tic suppression within the stress condition. The Friedman test showed that the difference between the three situations reached trend level (*p* = 0.099). *Post hoc* tests were run with Wilcoxon signed-rank tests: The salivary cortisol level during stress was higher as compared to concentration (*p* = 0.011), but there was no significant difference between the salivary cortisol level during stress and relaxation (*p* = 0.117). A Wilcoxon signed-rank test also showed that there was no significant difference between the stress + tic suppression condition and the stress + no suppression of tics condition (*p* = 0.263).

### Heart Rate

There was a main effect of situation on the heart rate [*F*(2,30.88) = 79.67, *p* < 0.001]. *Post hoc* tests revealed that all situations differed (*p* < 0.001) with the heart rate being highest during stress and lowest during relaxation. The main effect of instruction reached trend level [*F*(1,25) = 4.01, *p* = 0.056]. There was no interaction between the factors situation and instruction.

### Number of Skin Conductance Responses

Situation had a main effect on the NSCR [*F*(2,48) = 16.86, *p* < 0.001]. *Post hoc* tests revealed that this effect was driven by a higher NSCR during stress compared to both concentration (*p* < 0.001) and relaxation (*p* < 0.001). There was no effect of instruction on the NSCR and no interaction between the factors situation and instruction.

**Figure 3:**
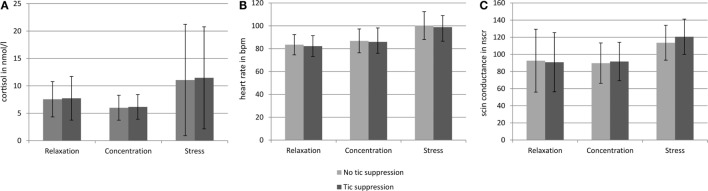
**Salivary cortisol level, heart rate, and skin conductance in the six different conditions**. Error bars indicate the SDs. **(A)** Indicates the cortisol concentration in the saliva in nanomoles per liter obtained from the samples taken at the end of each of the six conditions. **(B)** Indicates the heart rate in beats per minute (bpm) during each of the six conditions. **(C)** Indicates the number of skin conductance responses (NSCR) during each of the six conditions. For the number of tics, see Figure 2 and Table 1.

Figure 3 gives an overview of salivary cortisol, heart rate, and skin conductance in the three different situations.

In the relaxation and the stress condition, there was no correlation between the three different measures of biological stress, but in the concentration condition there was a positive correlation between cortisol and heart rate (*r* = 0.045, *p* = 0.023).

### Perceived Stress Scale

There was a main effect of situation on the rating of perceived psychosocial stress [*F*(2,27.47) = 25.34, *p* < 0.001]. This effect was driven by higher ratings for the stress situation compared to both concentration (*p* = 0.001) and relaxation (*p* < 0.001). There was no effect of instruction and no interaction effect of situation and instruction on the perceived psychosocial stress.

### Self-Assessment-Manikin Scale

There was a main effect of situation on the subjective pleasure rating [*F*(2,19.82) = 15.91, *p* < 0.001]. *Post hoc* tests revealed that this effect was driven by lower pleasure ratings for the stress situation compared to both concentration (*p* = 0.003) and relaxation (*p* = 0.002). There was no effect of instruction and no interaction effect of situation and instruction on the pleasure rating. There was also a main effect of situation on the subjective rating of arousal [*F*(2,34) = 17.86, *p* < 0.001], which was driven by higher arousal ratings for the stress situation compared to both concentration (*p* = 0.002) and relaxation (*p* < 0.001).

### Premonitory Urges

There were no effects of situation or instruction on the rating of premonitory urges as obtained with the PUTS. However, the main effect for situation reached trend level [*F*(2,32) = 2.66, *p* = 0.086]. *Post hoc* tests revealed that this trend was driven by trend for higher urge ratings in the stress situation compared to the relaxation situation (*p* = 0.059).

### Correlations between YGTSS Scores and Number of Tics

The severity of motor tics at baseline (as measured with the YGTSS) was positively correlated to the number of tics in all of the six conditions [correlation coefficients ranging from *r* = 0.445 (*p* = 0.016) to *r* = 0.554 (*p* = 0.001)]. There was no correlation between baseline severity of vocal tics and the number of tics during the experiment. The total tic severity score was positively correlated to the number of tics during the relaxation conditions [relaxation + no suppression of tics: *r* = 0.370 (*p* = 0.040), relaxation + tic suppression: *r* = 0.367 (*p* = 0.046)]. There was no correlation between total tic severity at baseline and the number of tics during concentration and stress.

### Correlations between Biological Measures of Stress and Affective Reaction

We did not find correlations between cortisol, heart rate, or skin conductance and scores of the PSS-4, SAM, or PUTS in any of the conditions.

## Discussion

The aim of our study was to investigate the effect of psychosocial stress on short-term tic fluctuations in children and adolescent with TD. To the best of our knowledge, this is the first study using a standardized method to induce psychosocial stress, using physiological measures of stress to validate the stress induction and using objective measures of tic frequency in parallel.

We video recorded the number of tics during a standard stress induction task and compared it to the tic frequency during situations in which the participants were relaxed or concentrated. In order to analyze the effect of stress on the ability to suppress tics, we gave our participants two different instructions for each situation: once they were asked to suppress their tics and once they were asked to “tic freely.”

During the stress induction task, we observed clearly the expected increase of salivary cortisol, heart rate, and NSCR. Accordingly, the subjective rating of perceived psychosocial stress was highest during the stress induction task, as compared to both other situations concentration and relaxation. In addition, the stress situation was rated less pleasant and more arousing than both other situations, i.e., concentration and relaxation. These findings are in line with previous studies using the TSST-C (20, 26–28) and prove that our participants were effectively stressed by the task, irrespective of the instruction to suppress their tics.

Our main variable of interest was the number of tics. In general, the number of tics was lowest during stress and highest during relaxation, and there were fewer tics when the participants were instructed to suppress them. However, the most important finding is the interaction between the factor situation and the factor instruction: there was a clear effect of instruction during relaxation and concentration. As expected, the participants exhibited a lower number of tics, when instructed to suppress them. However, the instruction to suppress the tics did not have any effect during the stress induction task. In both stress conditions, the number of tics was equally low, i.e., with or without instruction to suppress them, and similarly low as in the other conditions (relaxation and concentration) with the instruction to suppress the tics (see Figure 2).

At first glance, these findings speak against previous suggestions that stress leads to a short-term increase of tic frequency (10, 11, 14). Our findings are also not fully in line with recent studies on the role of the autonomic nervous system in Tourette syndrome (29). In a skin response biofeedback study, tics were lower during relaxation biofeedback compared to arousal biofeedback (30). However, those previous studies differ substantially from ours with regard to the experimental design that has been used and with regard to the methods that were used to measure stress and tic frequency, making it difficult to draw comparisons.

Interestingly, results of Wood et al. (15) on short-term changes in tic frequency determined also from video recordings are mostly in line with ours. In this study, patients (*n* = 4) with TD watched emotional scenes from a movie. The tic frequency was consistently lower during emotionally charged scenes than during baseline and especially low during happy and anger scenes (15). This corresponds to our finding that tic frequency was lower during stress compared to relaxation and concentration, to the effect that both findings suggest that situations with strong emotional valence (i.e., happy and anger scenes in the study by Wood et al. and the stress induction task in our study) might have a tic suppressing effect, at least on a short-term perspective.

But how does this fit together with the self-report studies and experts’ statements about increases of tic frequency in response to elevated levels of psychosocial stress? (5–8). A possible explanation might be the subjective experience of a rebound effect after tic suppression independently of a stress level. Most recent studies argue against a rebound effect (31–35) by reporting that tic frequency solely returns to baseline level after a period of tic suppression but does not exceed that baseline level. However, due to difficulties in rating their own tic frequency validly (32), patients might perceive this differently. Conceivably, the patients might mistake the post-suppression increase of tics as an increase from baseline level and attribute it to the preceding suppression situation. In this way, they might report a stress-related increase of tics, when tic frequency solely goes back to baseline after a period of suppression during stress.

Beyond the assumption that stress itself might have a suppressing effect on tic frequency, the level of focused attention on the stress induction task might have been the key component responsible for the observed tic reduction. It speaks against this alternative explanation that tic frequency was not reduced in the concentration situation, in which the patients were also attentive but not stressed. However, one might argue that the participants’ children have concentrated more during storytelling than during the symbol search task, because they were more motivated to concentrate in this stressful situation.

A recent study has indicated that psychosocial stress does not increase short-term tic frequency *per se*, but that stress increases the tic frequency, because it mainly reduces the ability to suppress tics (16). In order to take this possibility into account, we included a reinforced tic suppression condition in the stress induction task and in both the concentration and relaxation situations. Since we found that the tic frequency during reinforced suppression in the stress induction task was similarly low as in the other situations during reinforced suppression, we cannot completely support the suggestion by Conelea et al. (16). However, since reinforced tic suppression did not have any effect during the stress induction task, we can confirm that the ability to suppress tics is reduced, when a patient with TD is under stress. The difference between the previous findings (16) and our results might also be explained by the different types of stress induction used. While Conelea et al. (16) used a math task, which required cognitive and attentional effort that might have influenced tic suppression independent of the individual stress level, the free speech task does not put such high demands on both cognition and attention.

The current study has several limitations that have to be taken into account. First, with the free speech task, we induced a specific type of stress, i.e., psychosocial stress. Thus, our results might not be generalized to other types of stressors. Second, we induced the intention to suppress tics by instructing our participants to do so and by reinforcing successful suppression, but without getting feedback about the individual effort they put in the tic suppression. We, therefore, could not rule out that the low number of tics in the stress situation might be due to an (uninstructed) increase of the participant’s suppression effort. Third, we also do not know how well the symbol search task worked in inducing concentration, because we did not collect the outcome measures of this task to avoid a “stress-inducing component.” In order to be able to further analyze the differential effect of attention on the number of tics, future studies might include a dimensional and precise measure of concentration that does not induce stress at the same time. Furthermore, as mentioned above, since the stress situation might have included an inherent need for concentration, it is impossible to completely rule out concentration as a potential driving force for the reduction of the tics. It would therefore be interesting to see whether future studies using a form of stress situation that does not require as much concentration as the present test would obtain similar results. Finally, our study only focuses on the short-term fluctuation of tics. It would be an interesting question to address in future studies, if those stress-related short-term fluctuations are related to the long-term fluctuations of tics.

## Author Contributions

Judith Buse and Veit Roessner planned and designed the study. Stephanie Enghardt ran the data acquisition. Judith Buse and Stephanie Enghardt an the statistical analysis. Judith Buse wrote the first draft of the manuscript. All authors critically reviewed the manuscript.

## Conflict of Interest Statement

The research was conducted in the absence of any commercial or financial relationships that could be construed as a potential conflict of interest.
